# Durability Modeling Review of Thermal- and Environmental-Barrier-Coated Fiber-Reinforced Ceramic Matrix Composites Part I

**DOI:** 10.3390/ma11071251

**Published:** 2018-07-20

**Authors:** Ali Abdul-Aziz

**Affiliations:** Department of Aerospace Engineering, College of Aeronautics and Engineering, Kent State University, Kent, OH 44242, USA; aabdula3@kent.edu

**Keywords:** composites, ceramics, EBC, TBC, failure modes, modeling, coatings, materials testing, processing

## Abstract

This paper is a Part I of a literature review documentation describing the currently available and used techniques that are being explored by material scientists and researchers in the field of materials characterizations and testing for both thermal and environmental barrier coatings (TBCs and EBCs, respectively). This review contains relevant information regarding the most common coating applications and their impact on the durability and life of both the coatings and the substrate materials. It also includes a description of the methodologies of coating applications and their pros and cons. A discussion of the applicability, failure modes and modeling approaches that are presently available and utilized by active researchers in the field is also included. Part II will illustrate an in-depth assessment of various aspects of the available and developing life prediction models for both TBC and EBC and the influence of intrinsic and extrinsic factors on their thermal and mechanical stability.

## 1. Introduction

Thermal barrier coatings (TBCs) were developed about three decades ago to be used by engine manufacturers for improving engine performance and fuel efficiency to allow for higher operating temperatures while reducing emissions and costs, increasing the life-time of an engine and reducing maintenance costs. However, upon exposure to hot gas and harsh environmental conditions, they tend to undergo some types of degradation phenomena [1] which considerably impact their effectiveness. Therefore, research to improve their durability and performance is actively ongoing [2]. Nevertheless, other types of protective coatings mainly for ceramic matrix composite (CMC) substrates, such as environmental barrier coatings (EBCs) are evolving to compete with TBCs to offer an environmental shield for the hot section components of the turbine engine. However, in service, the structure and composition of the various layers change due to engine operation and environmental conditions. These conditions can be attributed to sintering of the substrate layer, oxidation of the bond coat, and inter-diffusion phenomena with the substrate. As a result, the properties of each layer are affected, as is the interfacial toughness. These progressions, combined with applied external stresses, may lead to bond coat deterioration, crack formation at the bond coat–ceramic interface and the ceramic layer may eventually spall off. In addition to these inherent degradation modes, interactions with the environment can expedite system degradation. 

Thus, evaluating coated components and subcomponents made out of monolithic and composite ceramics under gas turbine engine conditions is highly essential to demonstrate that these materials will perform as expected and required under these aggressive environmental circumstances. 

**Figure 1:**
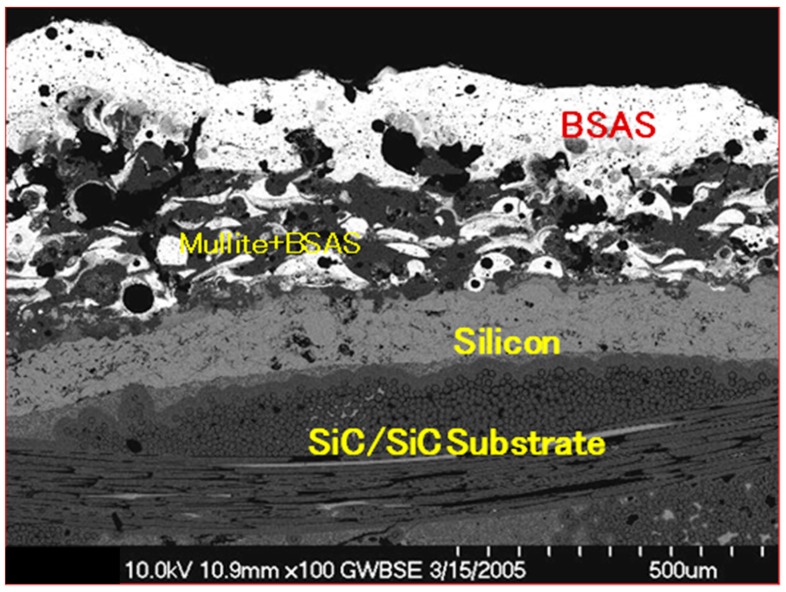
SEM micrograph of a typical cross-section of a plasma-sprayed environmental barrier coating (EBC) on a melted infiltration (MI) SiC/SiC substrate showing the microstructure, composition, and thickness of EBC sublayers.

The EBC system is made up of multiple layers of coatings in which each layer serves a specific purpose (Figure 1). The composition, total thickness, and processing methods can vary depending on the component type and intended life requirements of the coating. They consist of a bond coat and two or more layers of top coats depending on the functionality required. The bond coat facilitates the adherence of the ceramic top coat to the substrate and the ceramic top coat offers protection from water vapor and thermal insulation. Key requirements for a successful EBC include [3], environmental stability, especially in water vapors; and the reduction of oxygen and moisture diffusion to the substrate. It is also desirable to have low thermal conductivity for maximum thermal insulation capability.

The TBC is comprised of two layers: a metallic bond coat and a ceramic top coat [4]. The bond coat has two functions: it offers a bonding mechanism between the top coat, which is typically made out of ceramic, and the substrate like a superalloy or composite materials. This will lead to protecting the substrate from any environmental degradation.

Additionally, a reaction product identified as a thermally grown oxide (TGO) develops and grows at the TBC-ceramic interface due to high temperatures. The thermal expansion coefficients of these constituents of the TBC system are different and as a result, this leads to thermal stresses mismatch due to the existence of the temperature gradient in the structure. This causes stress growth in the TGO section due to the incessant formation of TGO due to the presence of high temperature. 

The stresses in the TBC are affected by the surface roughness of the bond coat and both the TGO growth and the thermal expansion coefficient (CET) mismatch stresses are compressive on flat bond surfaces [5].

The mismatch thermal stress (σ_t_) is given by Equation (1),

σ_t_ = (α_c_ − α_substrate_) ∆T E_c_/(1 − ν_c_) (1)

where α_c_ and α_substrate_ are coefficients of thermal expansion for the coating and the substrate, E_c_ is the Young’s modulus of the coating, and ν_c_ is the Poisson’s ratio of the coating.

**Figure 2:**
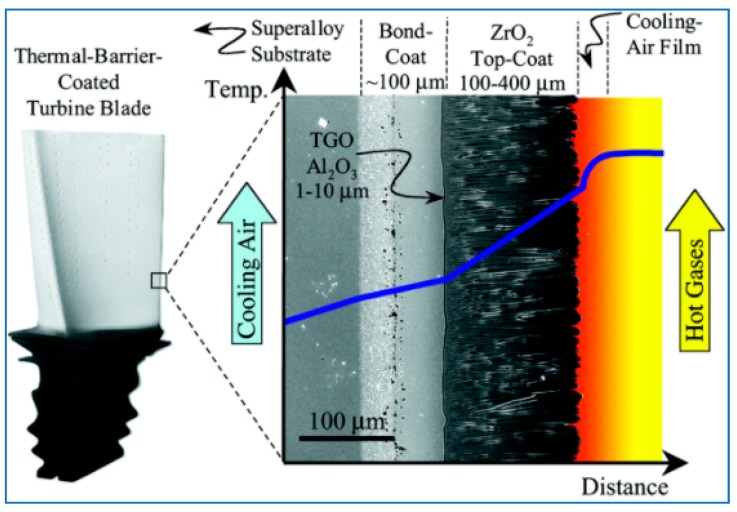
Cross-sectional SEM micrograph of an electron-beam physical-vapor-deposited (EB-PVD) thermal barrier coating (TBC) superimposed onto a schematic diagram showing the temperature reduction provided by the TBC. The turbine blade contains internal hollow channels for air-cooling, whereas the outside hot-section surface is thermal barrier coated, setting up a temperature gradient across the TBC. *(1) TBCs for Gas-Turbine Engine Applications*. Available from (accessed 1 June 2018). [6].

Figure 2 shows a reprehensive layout of a TBC application over a turbine blade that contains internal hollow channels for air-cooling, whereas the outside hot-section surface is thermal barrier coated, setting up a temperature gradient across the TBC [6]. The key function of the ceramic top coat is to reduce the alloy surface temperature by insulating it from the hot gas.

Thermal barrier coating (TBC) applications remains attractive as the need for greater engine efficiency in aircraft and or improving engine performance is the desire of all engine makers. They further prefer to be able to operate at higher operating temperatures while reducing emissions and costs, increasing the lifetime of an engine, and reducing maintenance costs [7]. However, durability issues tend to affect the benefits that can be obtained from using TBCs [5,7]. Thus, the need for a full understanding of TBC failure phenomena is essential for predicting its service life.

## 2. TBC and EBC Physical Characteristics

Table 1 shows the material characteristics that are relevant to the modeling of both TBCs and EBCs. A clear difference in their durability tolerance and application is very obvious from the information and data shown in the table. 

**Figure 3:**
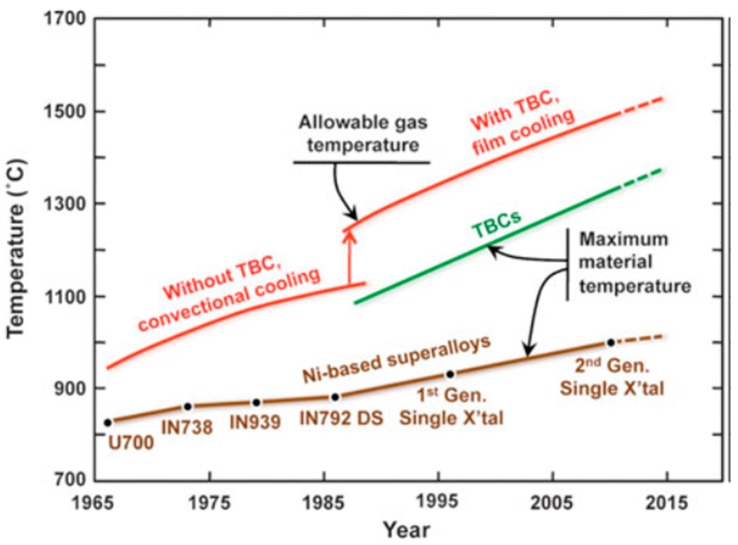
Progression of temperature capabilities of Ni-based superalloys and thermal-barrier coating (TBC) materials over the past 50 years. The red lines indicate progression of maximum allowable gas temperatures in engines, with the large increase gained from employing TBCs. Based on a diagram from the late Professor Tony Evans [2].

Figure 3 shows an illustration of the temperature capabilities of the TBC compared to Nickel based superalloys. As noted, the TBC is able to withstand an increase of temperature from nearly 1100 °C to 1500 °C with and without film cooling. This an indication that having such coating materials available to protect engine components has tremendous effects on engine capabilities and performance [1]. This further indicates that the gas temperature increase triggered by the application of TBC combined with an innovative cooling technique, has led to much greater temperature capabilities compared to earlier materials development including the single-crystal NI-based superalloys.

## 3. TBC and EBC Processing Methods

Generally, both TBCs and EBCs are coating materials that are being considered for as protective systems for CMCs for components used in aeroengine applications. CMCs are materials that are lighter and are able at a temperature that is 200 °C hotter than that is needed for the metallic materials. A protective silica forms in dry air conditions on the surface, which provides stability at temperatures up to 1300 °C for long-term situations. However, in combustion environments with presence of moisture, the silica layer on surface reacts violently leading to surface recession [8,9]. Therefore, a protective system is very critical for CMC to perform efficiently in aero-engine harsh and complex operating environment. Avoiding such recession is currently being repelled by the introduction of a variety of EBCs systems. These materials have been undergoing development over the last 15 years [10,11,12,13,14].

Currently, these systems are deposited by various methods, the most popular of which include: (a) air plasma spray (APS); (b) electron-beam-assisted physical vapor deposition (EB-PVD); (c) sputtering; (d) slurry deposition; (e) plasma spray assisted physical vapor deposition (PS-PVD) and (f) chemical deposition process (CVD). The microstructure, elastic and fracture properties of the coating deposited by the above methods can vary significantly. Among the most successful application to apply, EBC is the APS [15]. With the EP-PVD process, due to the low vapor pressure of silica in comparison to both the alumina and rare earth oxides, it makes problematic to produce coatings at an optimum level of stoichiometry. There are other coating application procedures being considered such as chemical vapor deposition (CVD) [16], sol-gel, and slurry coatings [17]. Listed in the following subsections description of most used deposition applications. 

### 3.1. Plasma-Sprayed Coatings

Currently, among the preferred method for producing TBCs and EBCs is the plasma-spray process. This process is generally initiated by briefly filling ceramic or metallic powder into and arc plasma at high velocity and at high temperature. As this process proceeds, the powder particles melt as they are pushed towards the substrate and as a result, the molten materials forms over the substrate to build up the coating level desired. Parameters controlling the quality of the coating have to be optimized such as productivity of good coating is expected. Also, careful monitoring of the process is required to guarantee reproducibility.

**Figure 4:**
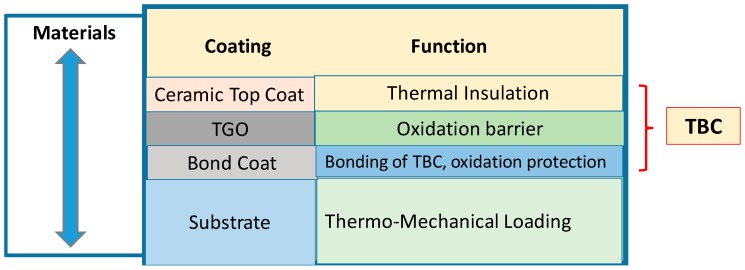
Scheme of coating construction of TBC barrier layers and the roles of the individual sublayers.

Figure 4 represents a scheme arrangement of a coating of TBC barrier layers showing the individual sub-layers and their role [18]. As seen, typical coatings materials constituents are noted as well as their systematic sequence. The ceramic top coat is used as a thermal insulator followed by TGO as an oxidation barrier and lastly by the bond coat serving as oxidation protection. The nickel base super alloy is the substrate that is generally subject to thermomechanical loading. 

Figure 1 shows a detailed representation of a typical coating layout for a SiC/SiC composite substrate specimen SEM micrograph cross-section showing the coating layers’ sequence and substrate materials. It is further obvious that micro-structural irregularities such as voids, porosities and pre-cracking are clearly seen and eminent. Additional facts are also noted about the distribution of the coating and the range of porosities and impurities involved in the coating application process. Such discrepancies and non-uniformity in the distribution constitute a lead cause for cracking and other structural abnormalities of the coating system. Further, in this particular specimen, Figure 1, the top coat is made out of barium aluminum strontium (BSAS), the intermediate coat is made out of BSAS and mullite, and the bond coat is made out of silicon. 

### 3.2. The Electron-Beam Physical Vapor Deposition (EB-PVD) Process

The electron-beam physical vapor deposition (EB-PVD) process is another application used to prepare ceramic barrier coatings. During the application of this process, high-energy beam is applied to heat and vaporize the ceramic. The vapor moves under direct line of sight to substrate and it forms as atom-by-atom layer. If zirconia—yttria are used as a ceramic material, the process of application is applied under low partial pressure of oxygen so the stoichiometry of the zirconia is preserved. 

Application of EB-PVD to deposit costing leads to an interface between the bond coat and the ceramic. Note in the application, the ceramic has a smooth surface while for plasma-sprayed coatings a ceramic rough surface is required. After deposition of an initial thin region, the ceramic exhibits a columnar growth. After deposition of an initial thin region, the ceramic displays columnar growth. This growth is known to help reducing the elastic modulus along the plane direction of the coating close to zero. This resulted in better lifetimes for the TBC deposited via EB-PVD on cylindrical shape substrate [19]. Nevertheless, the coating complex geometries continues to affect its re-productivity and viability of its application.

### 3.3. Sputter-Deposited Thermal Barrier Coating

Sputter-deposited thermal barrier coatings have been researched in very widely for longtime [17]. Sputtering is a process, which takes place when a negatively charged target is blasted with ions thereby instigating the target material to be expelled. The expelled material then lays down positively charged substrate. The benefit of sputtering is that it operates at a low temperature, which will enable the production of a wide variety of microstructures. This includes columnar structure that is very similar type that produced by the EB-PVD process. Production of TBC with properties impending those of a plasma-sprayed coating has yet to be possible [17].

### 3.4. Processing of Plasma-Spray Physical Vapor Deposition (PS-PVD) TBC Coating

The PVD system requires a multiple step coating sequence process with precise controls. The function of the bond coat is to provide a stable base for adherence of the ceramic insulation layer. The bond coat must generate a stable, slow-growing alumina scale. In the PVD process the coating material is heated to vaporization temperatures through kinetic energy exchange between an electron beam (~38 kV) and the coating material [20]. The process is highly specialized such that large amounts of energy can be focused in a small area and evaporation can be obtained by sweeping the beam over the surface of the material. Ceramic materials like ZrO_2_-7%Y_2_O_3_ have very high melting points (=2700 °C) and very low thermal conductivities. 

It is estimated that the evaporation temperature at the point of electron beam impact is >5000 °C [21,22,23]. The coating material evaporates atomistically (or molecularly) and condenses onto the part. The atoms move on the surface in their condensed state and form nuclei to begin grain growth. Columnar growth is the typical norm; however, grain orientation can be influenced by the processing temperature and surface condition. In order to obtain a good PVD TBC coating, the parts must be processed at high temperatures. The formation of the coating is dependent on the melting point of the coating material and the substrate being coated [21,22,23]. For instance, having a part with very close temperature to the melting point of the coating materials, this will result in having good surface mobility of the condensed atoms and thus a denser coating is produced. 

### 3.5. Slurry Deposition Technique

The slurry spray technique utilizes traditional wet powder spraying methods to deposit sinterable coating materials onto target substrates to be sintered to produce a functional coating. The process includes liquefying the coating material to form a slurry mixture that can be applied to a surface using a standard spray gun. Layers are sprayed in a sequential format onto the substrate. Then dried using different slurry compositions. The preferred thickness of the layers to prevent surface cracking during the drying process is approximately 0.25 mm and the drying time is about an hour, depending on the environmental conditions. After the desirable number of layers of the TBC is created, the multi-layered coating is pressed in a compression chamber to create a solid form before being sintered with an acetylene torch or furnace. 

The applied pressure varies depending on the number of coating layers, typically between 10 and 40 MPa [24]. This coating technique has many advantages compared to other coating methods. It is very simple to use which reduces fabrication costs, and it has the potential to be used on a wide range of coating materials. Provided that they can be prepared as a powder capable of being sintered at temperatures below the melting point of the substrate as well as the ability to coat complex surface geometries. The process has the potential to be adopted in an automated environment but can also be utilized for manual applications. The procedure requires no inaccessible or emptied environments and can be utilized to coat bigger surface with relatively cost-effective rate compared to other coating applications [25].

### 3.6. Chemical Deposition Process (CVD)

CVD is being developed as a TBC forming technique because of its non-light-of-sight nature and excellent conformal coverage characteristics. During the CVD process, a zirconium precursor such as Zr (dpm)4, and a yttrium precursor such as Y (dpm)3, are sublimed. The vapors are transported into a mixing chamber by a carrier gas of Ar. The secondary gas of oxygen is added into the mixing zone above the substrate [3,4]. TBC deposition is performed at <±1000 °C. A columnar structure is formed. Thermal chemical vapor deposition (TCVD) and plasma-enhanced chemical vapor deposition (PECVD) can reach the deposition rate of about 200 µm/h, and laser-assisted chemical vapor deposition (LACVD) can reach a deposition rate higher than 600 µm/h, which is comparable to the deposition rates of APS and EB-PVD. LACVD is advantageous in preventing premature powder formation because the reaction site is restricted to a very small area on the substrate surface by a focused laser beam. A well-developed columnar structure produced in LACVD TBC is shown in Figure 1. The high deposition rate causes the formation of a large number of nano-pores in the LACVD TBC, leading to a small thermal conductivity of 0.7 W/(m·K).

It should be noted that there are other procedures capable of coating ceramic and metallic materials. These procedures are older flame spray guns and the newer ones such as detonation guns [26], water-stabilized plasma spraying and hypersonic spraying. However, the flame-spraying gun did not deliver satisfactory results when zirconia-based thermal barrier coatings was applied. Yet, it is expected that newer methodologies will arise and lead to much improved TBCs and better application process.

Nonetheless, these application methods have some flaws as well as advantages. For instance, the primary advantage of sputtering is its capability to deposit an extensive variety of materials (e.g., alloys, oxide solutions, and intermetallics).

However, sputtering is yet to be considered as production method used for turbine engine components. This because the available equipment used in this process deposits the coating in a very slow manner. The physical vapor deposition process is an atomistic deposition method that encompasses the vaporization and subsequent deposition of coating species. It has the capability of delivering deposition of coatings of metals, superalloys, and ceramics on most materials and on a variety of shapes. It is a simple system to use.

However, one deficiency in applying the traditional thermal spray techniques has been linked to having accurate deposition and thinness of the coatings. This makes the EB-PVD a preferred method in comparison to the plasma spray since a much smoother surface finished is attained as a result. Further, better strain tolerance, improved erosion-resistance properties, and better-quality metallurgical bonding with the substrate.

Although in terms of erosion resistance and strain compliance the EB-PVD TBC systems has significant advantages over plasma sprayed TBC’s, in many applications in the gas turbine engine, such as the combustion chamber and nozzle guide vane platforms, plasma sprayed TBC performs sufficiently. These applications for plasma sprayed TBC’s will remain being used and because of their relatively low cost compared to EB-PVD TBCs, their application to these types of components in the future is somewhat guaranteed. Since EB-PVD technology proofed to be more effective than plasma spraying when depositing TBC on the airfoils of the modem gas turbine engines, where a good surface finish is vital and excellent strain compliance and adhesion are required. In these areas, EB-PVD offers significant benefits over plasma spraying which justifies the higher manufacturing costs of the EV-PVD. Pros and cons of most common and popular deposition technologies [27] are shown in Table 2.

## 4. Testing of EBCs and TBCs

One of the major issue in EBC testing is the water vapor. Highly specialized laboratories with high steam capabilities [28] are used to simulate such environment. Meanwhile, high pressure/high velocity burner rigs [21] are used to imitate the high gas velocity and high vapor velocity. A very small value of gas velocity (cm/sec) is generally used as a scale in the high steam rigs during testing for simplicity purpose. Since water vapor is the parameter that affect the performance of the EBC the most, this makes such test very suitable to identify the most applicable candidates of EBC for evaluating long-term EBC response. The high pressure-high and high-velocity burner rig test is very useful as proof test for checking the maturity of the EBC. It further can be used to run tests on sub-components since actual gas turbine environment can easily be simulated. Additionally, burner rigs can be used to produce temperature profile with gradient through the EBC simulating cooling and heating effects simultaneously. Laser rigs are used for thermal conductivity measurements and for the assessment of EBC performance under a temperature gradient [29,30,31,32].

Prediction and Accuracy: Once all the material properties for the reinforced ceramics (SiC/SiC) and TBCs/EBCs are available, it should be possible to simulate a simply supported beam for temperature and simulate how there is degradation in different materials and which one will fail first. A set of mechanical tests adopted by the American Society for Testing and Materials (ASTM) for materials characterization and durability assessments can be found in reference [33]. These tests can be considered to quantify the materials behavior and the useful life for both TBCs and EBCs. Suitable tests that can be considered are: ASTM C1275 and ASTM C 1359–tensile testing at room and high temperature; ASTM C1358–Compression testing, ASTM C1292 and ASTM C1425–shear strength at room and high temperature. Additionally, if available the CTE variation with temperature change for all the materials (SiC/SiC, EBC, TBC, etc.) have to be accounted for in any analysis performed.

## 5. Failure Modes of TBCs and EBCs

Currently, under steady state conditions, the failure phenomenon of the EBCs are not fully understood and models to estimate or predict the life of the EBCs are not available either. Thus, the need to develop modeling methodologies to determine and assess the life of the EBC and its durability is very essential to its success. However, factors that affect or influence the EBC stability, mechanisms of degradation and failure modes are to be determined and included in the modeling process. Predicting the durability and the life of the EBC alone and the CMC after the coating failure is very crucial to introducing CMC engine components coated with EBC.

Hence, the need for modeling the elements that influence the performance of the EBC and the CMC is of high importance to engine makers and the aviation industry. In order for the CMC to be successful in the use of making engine components, the coatings that are applied have to offer a reliant means of protection. This translates into the fact that the life of the coated CMC is highly dependent on the life of the coating and not the uncoated substrate life. It further means that if the coating fails under certain condition, then then the CMC life is considerably decreased. This approach of design is in line with that of the TBC coated metal parts currently used in engines where the design life of TBC coated metal is purely dependent on the life of the uncoated part during engine operating conditions. 

**Figure 5:**
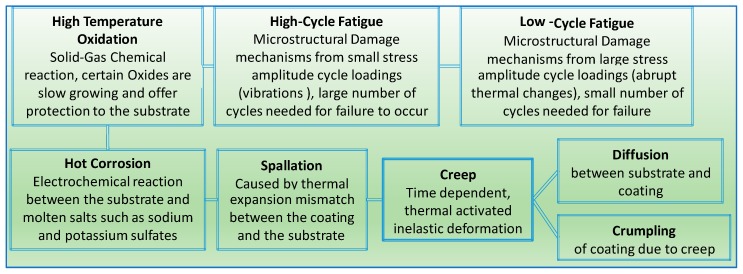
Chart of common failure mechanisms of TBCs and EBCs.

Among the most critical issues [31,32] that relate to the development of the EBC/TBC system is the chemical instability between silica formation, silicon-based ceramics and EBC. This results in the development of pores that lead to interface delamination. Furthermore, volatilization of the silica under high temperatures in the presence of moisture and oxygen as well as a mismatch between the CTEs of the substrate and coating results in the formation of residual stress and surface cracks. Also, other concerns, such as flaws due to foreign objects impacts, through and surface cracks due to thermo-mechanical loads, and sintering and spalling due to thermal cycling are worrisome. Moreover, the failure modes of EBCs and TBCs to some extent share some similarities (Figure 5). As noted, in the figure, the failure mechanism is clearly illustrated in a sequential manner. Also, the descriptive layout presented is based on data collected from testing and other practical outcomes typically experienced by the two the materials. For example, during the manufacturing process, several passes of the coating are applied under specified thermal conditions that involve heating and cooling cycles which result in residual thermal stress depositions. This process leads to micro level cracks in the surface as well as through thickness ones. In addition, other factors lead to more cracks in the coating surface, such as foreign body objects and the thermal gradient between the substrate and the coatings, which are typically caused by thermal shocks and mismatch in the material’s thermal properties. 

Other issues such as oxidation of the bond coat have been frequently identified as important factors affecting ceramic top-coat durability during service [33,34,35]. The major outcome due to oxidations is the spalling of the coating which is also due to other factors that accompany bond coat oxidation such as volumetric expansion during high temperature exposure. Oxidation occurs due to the penetration of water vapor from the combustion gases through the EBC/TBC and top-coat layers. Until now, the oxidation behavior with respect to the failure of the TBC remains to some extent not fully established. As a result, bond- and top-coat-related durability issues might lead to diminishing the TBC thermal fatigue life, both separately and through interactions with the mechanism of oxidation. These issues include bond coat coefficient of thermal expansion (CTE), bond coat roughness, and the creep behavior of both the ceramic and bond coat layers [34,35,36,37,38]. A study by Chang et al. [39] pointed out that oxidation has a very strong effect on the stress state of ceramic. Their work further led to the development of a mode to predict the yield stress of the bond coat, which included plasticity without oxidation and creep effects [40]. However, their models excluded the effect of creep of the constituents, multiple thermal cycles, or the progressive growth of the oxide over several cycles. Petrus and Ferguson [37] covered creep but they skipped including multiple thermal cycles or oxide growth. 

Consequently, research to improve TBC durability and performance remains actively ongoing including a better understating of its service life [41]. In the meantime, other types of protective coatings for CMC substrates such as EBCs are evolving to compete with TBCs to offer an environmental shield for the hot section components of the turbine engine. However, in service, the structure and composition of the various layers change, due to engine operation and environmental conditions. These conditions can be related to several factors, such as sintering of the substrate layer, oxidation of the bond coat, and inter-diffusion phenomena with the substrate. As a result, the properties of each layer are affected, as is the interfacial toughness. These developments, combined with applied external stresses, may lead to bond coat damage and cracks at the bond coat substrate interface. Such concerns might cause spallation and degradation due to interactions with the environment. The upper-use temperatures of these EBCs vary between 1200 and 1650 °C depending on the composition [41,42,43,44]. These coatings also recess with time at temperatures >1400 °C, but the rate of recession is an order of magnitude or two lower than that of the uncoated substrate at the same temperature.

**Figure 6:**
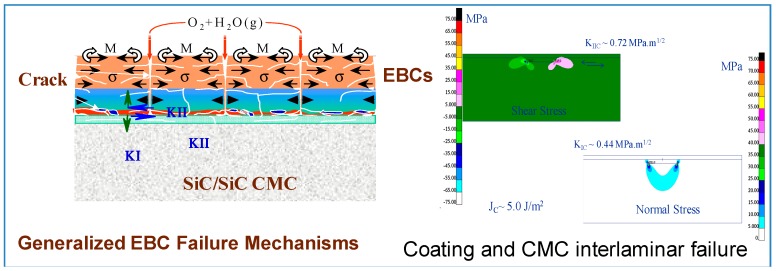
Failure mechanisms in EBC.

**Figure 7:**
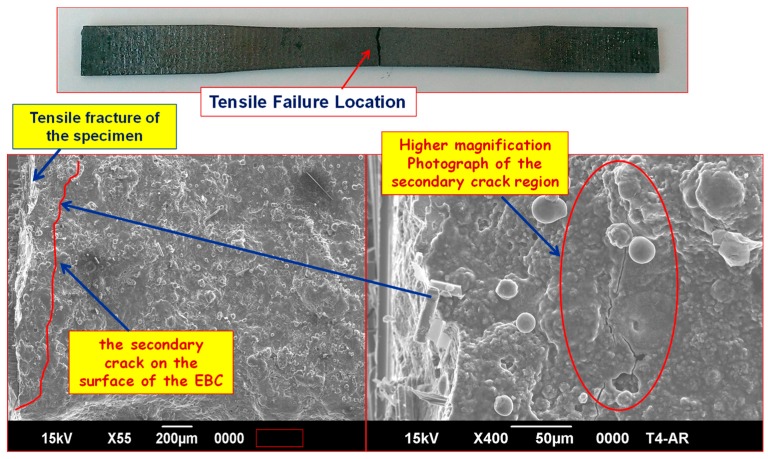
SEM photographs of the tensile-fractured specimen showing secondary cracks on the surface of the EBC.

Under therno-mechanichal cycling and due to foreign object damage, EBC can spall-off from the substrate. The failure mode can vary based on the operating conditions. Under thermal cycling condition without stress, two failure modes have been observed [43,44]:

(1) Cracks spanning from the top coat through the thickness to the bond/intermediate coat interface and to the bond/substrate line with a formation and growth of horizontal crack alongside the interfaces. These cracks are linked and eventual spallation of the coating is imminent. Also, reactions at the interface and the formation of internal pores further accelerate the coating spallation. Figure 6 shows the inter-laminar failure of the coating and the stress contour due to combined thermal and mechanical load obtained from a finite element based analysis.
(2) Moisture through the coating to the substrate/bond coat interface and oxygen diffusion followed by formation of pores and oxidation of the substrate, linkage of the pore and eventual spallation of the coating. This observation is noted when a mismatch of thermal expansion between the substrate and the coating co-exist. An example of this mechanism is shown in Figure 7, which shows pore formation at the substrate/coating interface leading to coating delamination.

In the as-processed state, TBCs are well-bonded to the metallic substrate upon which they are deposited. However, as these coatings are exposed to high temperatures during turbine operation, they lose their adhesion. Therefore, in order to assess the durability of the TBC, certain tests have to be performed and developed to monitor and determine how these testing techniques, coupled with fracture mechanics principles can track the loss of adhesion and identify the mechanisms causing it.

Tests are performed exposing the TBC to a variety of thermal exposures, including mechanism-based tests for isothermal dry air exposures, mechanism-based tests for exposures with water vapor and mechanism-based tests for cyclic thermal exposures. Therefore, TBCs and EBCs are more like monolithic ceramics (without fibers) and they do not need to be reverse engineered to predict the thermo-mechanical properties. They simply can be treated like metal (isotropic) and test data can be used to estimate the E, v, and strengths (ST, SC, SS). Besides mechanical tests, the CTE variation of all the materials (SiC/SiC, EBC, TBC, etc.) with temperature, change has to be determined.

Figure 7 shows the gage section of a failed dog bone specimen that was examined under a SEM [45]. A secondary crack formed on the EBC close to the fracture end. The opening of this crack is <<4 μm. In this situation, due to the poor depth of focus of the optical microscope, matrix cracks could not be resolved. SEM can resolve the crack only in a failed specimen very close to the fracture surface [45].

## 6. Conclusions

Assessing the failure of TBCs and EBCs is not a simple task, and data from rigorous testing under severe and typical operating environments where factors that influence the characterization of these materials are needed. Thus, this paper offers a review and a detailed description of the materials features for both TBCs and EBCs citing in-depth discussion of their processing, applications and failure modes. Comparisons between the two coatings, their applicability, and the difference in their durability tolerance showing the substantial variance is demonstrated in a tabular format. Further, ample information about their materials characteristics and methods of developments and applications were recited and described in a rather detailed manner. Listed in the bullet points below are some fundamentals regarding the development of more conclusive life models for both EBCs and TBCs.

(1) The need to develop modeling methodologies to determine and assess the life of the EBC and its durability is very essential to its success. However, factors that affect or influence the EBC stability, mechanisms of degradation and failure modes are to be determined and included in the modeling process.
(2) In order for the CMC to be successful in the use of making engine components, the coatings that are applied have to offer a reliant means of protection. This translates into the fact that the life of the coated CMC is highly dependent on the life of the coating and not the uncoated substrate life. It further means that if the coating fails under certain condition, then then the CMC life is considerably decreased.
(3) Predicting the durability and the life of the EBC alone and the CMC after the coating failure is very crucial to introducing CMC engine components coated with EBC.
(4) Finally, a detailed representation of TBC and EBC physical characteristics along with their processing methodologies, testing procedures/approach and failure modes has been summarized. The focus on their failure modes and in particular the EBC is an essential element of this review since it is quite complex and modeling such aspects of failure modes requires extensive analytical/experimental testing efforts to include the causing factors. However, this is outside the scope of this article.

## Figures and Tables

**Table 1:** Characteristic differences between TBC and EBC.

Thermal Barrier Coating (TBC)	Environmental Barrier Coating (EBC)
Applied and used in hot gas engine environment on metal, their role is to protect the engine components in the hot gas path from the effects of the operating temperature.	Used on ceramic matrix composites (CMCs) or for any high temperature application where oxygen is present, it is to help decreasing oxidation-induced recession in silicon-based ceramic composites, and a need to lessen the operating temperature to weaken creep in oxide-based composites.
An aero TBC is a zirconia-yttria (or other zirconia based) ceramic on a metallic MCrAlY bond coat over a superalloy. MCrAlY coatings (where M = Co, Ni or Co/Ni) are widely applied to first and second stage turbine blades and nozzle guide vanes, where they may be used as corrosion resistant overlays or as bond-coats for use with thermal barrier coatings.	An EBC or EBC/TBC has a zirconia or hafnia TBC top coat for thermal insulation over a silicate-ceramic environmental coating to protect the substrate from water-vapor attack.
Because of temperature, a buildup of a layer called thermally grown oxide (TGO) is generated between the ceramic layer and the bond coat. This is due to the oxidation effect of the bond coat during oxidation and thermal shock. The TGO is to hinder the process of oxidation of the bond coat.	A silicon bond coat is between the EBC and the substrate, which is a CMC. EBC are for ceramic and CMC substrates. EBC role is to provide protection from environmental assault.
TBCs are for metallic substrates and provide thermal protection. The TBC top coat is in compression.	EBC top coat is in tension. Cracks are easy to form, but tension cracks in EBCs are not as damaging as compression cracks are in TBCs
Compared to EBCs, TBCs have better strain tolerance.	Both TBCs and EBCs require a bond coat on top of their substrate followed by a top coat on the surface. Bond coats may be multilayered.
The major trigger of failure in thermal barrier coating is the stresses, they generally initiated by bond coat oxidation, bond coat surface irregularities, yttria stabilized zirconia (YSZ) phase transformation, and YSZ sintering [5].	Environmental barrier coating failure is generally triggered by chemical reactions not stresses. They further lead to degradation and spallation. Lead life-limiting reactions are water vapor volatility of the surface layer, chemical reactions between various EBC layers, including silica TGO, and the oxidation of silicon bond coat. Therefore, EBC design should take into account the latter effects and ensure that chemical reactions are limited to minimum [5].
Typical thickness: 1–1.5 mil bond coat, 3–4 mil top coats.	Typical Thickness: 15 mil for combustor (rough coating), 5–10 mil for vane (smooth), 5 mil goal for blade (smooth).

**Table 2:** Summary of the benefits and limitations of the atomistic and particulate deposition methods [27].

Features	Evaporation	Sputtering Deposition	CVD	Electro Deposition	Thermal Spraying
Mechanism to produce deposition species	Thermal energy	Momentum transfer	Chemicals reaction	Solution	Flame or Plasma
Deposition rate	Moderate up to 750,000 A/min	Low	Moderate	Low to High	Very high
Deposition species	Atoms	Atoms/Ions	Atoms/ion	Ions	Droplets
Complex Shape	Poor line of sight	Good but non uniform	Good	Good	Poor resolution
Deposits in small, blind holes	Poor	Poor	Limited	Limited	Very Limited
Metal/alloy deposition	Yes	Yes	Yes	Yes	Yes
Refractory compounds and ceramics	Yes	Yes	Yes	Limited	Yes
Energy of deposits species	Low	Can be high	Can be high	Can be high	Can be high
Growth interface perturbation	Not normally	Yes	Yes	No	No
Substrate heating	Yes normally	Not generally	Yes	No	Not normally

## Nomenclature

EB-PVD	Electron-beam physical vapor deposition
TBC	Thermal barrier coating
EBC	Environmental barrier coating
CMC	Ceramic matrix composite
CTE	Thermal expansion
E	Young’s modulus
v	Poisson’s ratio
ST	Tensile strength
SC	Compressive strength
SS	Shear stress
PS-PVD	Plasma-spray-assisted physical vapor deposition
CVD	Chemical deposition process
PECVD	Chemical vapor deposition
LACVD	Laser-assisted chemical vapor deposition
BSAS	Barium strontium aluminosilicate
α_c_	Coefficient of thermal expansion of the coating
α_substrate_	Coefficients of thermal expansion of the substrate, respectively
E_c_	Young’s modulus of the coating
ν_c_	Possion’s ratio of the coating

## References

[1] Evans A.G., Mumm D.R., Hutchinson J.W., Meier G.H., Pettit F.S. (2001). Mechanisms controlling the durability of thermal barrier coatings. Prog. Mater. Sci..

[2] Clarke D.R., Oechsner M., Padture N.P. (2012). Thermal-barrier coatings for more efficient gas-turbine engines. MRS Bull..

[3] Bose S. (2007). High Temperature Coatings.

[4] Jacobson N.S., Fox D.S., Smialek J.L., Opila E.J., Dellacorte C., Lee K.N., Cramer S.D., Covino B.S. (2005). ASM Handbook.

[5] Padture N.P., Gell M., Jordan E.H. (2002). Thermal barrier coatings for gas-turbine engine applications. Science.

[6] Lee K.N., Fox D.S., Eldridge J.I., Zhu D., Robinson R.C., Bansal N.P., Miller R.A. (2003). Upper temperature limit of environmental barrier coatings based on mullite and BSAS. J. Am. Ceram. Soc..

[7] Lee K.N. (2000). Current status of environmental barrier coatings for Si-based ceramics. Surf. Coat. Technol..

[8] Richard C.S., Beranger G., Lu J., Flavenot J.F. (1996). The influences of heat treatments and interdiffusion on the adhesion of plasma-sprayed NiCrAlY coatings. Surf. Coat. Technol..

[9] National Materials Advisory Board Committee on Coatings for High Temperature Structural Materials (1996). Coatings for High-Temperature Structural Materials: Trends and Opportunities.

[10] Abdul-Aziz A., Bhatt R.T., Grady J.E., Zhu D. (2012). Environmental barrier coating (EBC) durability modeling: An overview and preliminary analysis. Process. Prop. Adv. Ceram. Compos. IV.

[11] Zhu D., Miller R.A. (2000). Thermal conductivity and elastic modulus evolution of thermal barrier coatings under high heat flux conditions. J. Therm. Spray Technol..

[12] Ramasamy S., Tewari S.N., Lee K.N., Bhatt R.T., Fox D.S. (2011). Mullite–gadolinium silicate environmental barrier coatings for melt infiltrated SiC/SiC composites. Surf. Coat. Technol..

[13] Jorgensen P.J., Wadsworth M.E., Cutler I.B. (1959). Oxidation of silicon carbide. J. Am. Ceram. Soc..

[14] Smialek J.L., Robinson R.C., Opila E.J., Fox D.S., Jacobson N.S. (1999). SiC and Si_3_N_4_ recession due to SiO_2_ scale volatility under combustor conditions. Adv. Compos. Mater..

[15] More K.L., Tortorelli P.F., Walker L.R. (2001). Effects of High Water Vapor Pressures on Oxidation of SiC-Based Fiber-Reinforced Composites. Mater. Sci. Forum.

[16] More K.L., Tortorelli P.F., Walker L.R., Miriyala N., Price J.R., van Roode M. (2003). High-temperature stability of SiC-based composites in high-water-vapor-pressure environments. J. Am. Ceram. Soc..

[17] Miller R.A. (1995). Thermal Barrier Coating Workshop.

[18] Auger M.L., Sarin V.K. (1997). The development of CVD mullite coatings for high temperature corrosive applications. Surf. Coat. Technol..

[19] Roode M.V., Ferber M.K., Richerson D.W. (2003). Ceramic Gas Turbine Design and Test Experience: Progress in Ceramic Gas Turbine Development.

[20] Schulz U., Leyens C., Fritscher K., Peters M., Saruhan-Brings B., Lavigne O., Caliez M. (2003). Some recent trends in research and technology of advanced thermal barrier coatings. Aerosp. Sci. Technol..

[21] Bhatt R.T., Choi S.R., Cosgriff L.M., Fox D.S., Lee K.N. (2008). Impact resistance of environmental barrier coated SiC/SiC composites. Mater. Sci. Eng. A.

[22] Haynes J.A., Lance M.J., Cooley K.M., Ferber M.K., Lowden R.A., Stinton D.P. (2000). CVD Mullite Coatings in High-Temperature, High-Pressure Air–H_2_O. J. Am. Ceram. Soc..

[23] Rigney D.V., Viguie R., Wortman D.J., Skelly D.W. (1997). PVD thermal barrier coating applications and process development for aircraft engines. J. Therm. Spray Technol..

[24] Movchan B.A., Demchishin A.V. (1969). The Physics of metals and metallography.

[25] Thornton J.A. (1974). Influence of apparatus geometry and deposition conditions on the structure and topography of thick sputtered coatings. J. Vac. Sci. Technol..

[26] Thornton J.A. (1977). High rate thick film growth. Ann. Rev. Mater. Sci..

[27] (1996). National Materials Advisory Board Commission and Engineering and Technical Systems.

[28] Kellermann D.C., Furukawa T. A theory of strongly orthotropic continuum mechanics. Proceedings of the 5th Australasian Congress on Applied Mechanics (ACAM 2007).

[29] Nguyen P., Harding S., Ho S.Y. (2007). Experimental studies on slurry based thermal barrier coatings. Eng. Aust..

[30] Fèvre M., Finel A., Caudron R., Mévrel R. (2005). Local order and thermal conductivity in yttria-stabilized zirconia. II. Numerical and experimental investigations of thermal conductivity. Phys. Rev. B.

[31] Jiang X., Liu C., Lin F. (2007). Overview on the development of nanostructured thermal barrier coatings. J. Mater. Sci. Technol..

[32] Robinson R.C., Smialek J.L. (1999). SiC recession caused by SiO_2_ scale volatility under combustion conditions: Experimental results, empirical model and I. J. Am. Ceram. Soc..

[33] (2010). American Society of Testing and Materials (ASTM) C28 Standards.

[34] Soechting F.O. (1995). Proceedings of the Thermal Barrier Coating Workshop, Cleveland, OH, USA, 27–29 March 1995.

[35] Lee K.N. (2000). Key Durability Issues with Mullite-Based Environmental Barrier Coatings for Si-Based Ceramics. Trans. ASME.

[36] Lee K.N., Fox D.S., Bansal N.P. (2005). Rare Earth Environmental Barrier Coatings for SiC/SiIC Composites and Si_3_N_4_ Ceramics. J. Eur. Ceram. Soc..

[37] Bose S., DeMasi-Marcin J. (1997). Thermal barrier coating experience in gas turbine engines at Pratt & Whitney. J. Therm. Spray Technol..

[38] Brindley W.J. (1997). Properties of plasma-sprayed bond coats. J. Therm. Spray Technol..

[39] Wortman D.J., Duderstadt E.C., Nelson W.A. (1989). ASME Paper 89-GT-134.

[40] Petrus G.J., Ferguson B.L. (1997). A software tool to design thermal barrier coatings: A technical note. J. Therm. Spray Technol..

[41] Chang G.C., Phucharoen W.A., Miller R.A. (1987). Current status of thermal barrier coatings—An overview. Surf. Coat. Technol..

[42] Lee K.N., Fox D.S., Robinson R.C., Bansal N.P., Krenkel W., Naslain R., Schneider H. (2001). Environmental Barrier Coatings for Silicon-Based Ceramics. High Temperature Ceramic Matrix Composites. High Temperature Ceramic Matrix Composites.

[43] Zhu D.M., Bansal N.P., Miller R.A., Bansal N.P., Singh J.P., Kriven W.M., Schneider H. (2003). Thermal Conductivity and Stability of HfO_2_-Y_2_O_3_ and La_2_Zr_2_O_7_ Evaluated for 1650 °C. Advances in Ceramic Matrix Composites IX.

[44] Zhu D.M., Miller R.A., Fox D.S. Thermal and Environmental Barrier Coating Development for Advanced Propulsion Engine Systems. Proceedings of the 8th AIAA/ASME/ASCE/AHS/ASC Structures, Structural Dynamics, and Materials Conference.

[45] Abdul-Aziz A., Wroblewski A.C., Bhatt R.T., Jaskowiak M.H., Gorican D., Rauser R.W. Assessment of NDE methods for detecting cracks and damage in environmental barrier coated CMC tested under tension. Proceedings of the SPIE 9436, Smart Sensor Phenomena, Technology, Networks, and Systems Integration 2015.

